# Influence of Culture Media on Biofilm Formation by *Candida* Species and Response of Sessile Cells to Antifungals and Oxidative Stress

**DOI:** 10.1155/2015/783639

**Published:** 2015-02-01

**Authors:** Isela Serrano-Fujarte, Everardo López-Romero, Georgina Elena Reyna-López, Ma. Alejandrina Martínez-Gámez, Arturo Vega-González, Mayra Cuéllar-Cruz

**Affiliations:** ^1^Departamento de Biología, División de Ciencias Naturales y Exactas, Universidad de Guanajuato, Campus Guanajuato, Noria Alta S/N, 36050 Guanajuato, GTO, Mexico; ^2^Centro de Investigaciones en Optica AC, Loma del Bosque 115, Col. Lomas del Campestre, 37150 León, GTO, Mexico; ^3^Departamento de Ingeniería Química, Electrónica y Biomédica, División de Ciencias e Ingenierías, Universidad de Guanajuato, Campus León, 37150 León, GTO, Mexico

## Abstract

The aims of the study were to evaluate the influence of culture media on biofilm formation by *C. albicans, C. glabrata, C. krusei,* and *C. parapsilosis* and to investigate the responses of sessile cells to antifungals and reactive oxygen species (ROS) as compared to planktonic cells. For biofilm formation, the *Candida* species were grown at different periods of time in YP or YNB media supplemented or not with 0.2 or 2% glucose. Sessile and planktonic cells were exposed to increasing concentrations of antifungals, H_2_O_2_, menadione or silver nanoparticles (AgNPs). Biofilms were observed by scanning electron microscopy (SEM) and quantified by the XTT assay. *C. albicans* formed biofilms preferentially in YPD containing 2% glucose (YPD/2%), *C. glabrata* in glucose-free YNB or supplemented with 0.2% glucose (YNB/0.2%), while *C. krusei* and *C. parapsilosis* preferred YP, YPD/0.2%, and YPD/2%. Interestingly, only *C. albicans* produced an exopolymeric matrix. This is the first report dealing with the *in vitro* effect of the culture medium and glucose on the formation of biofilms in four *Candida* species as well as the resistance of sessile cells to antifungals, AgNPs, and ROS. Our results suggest that candidiasis *in vivo* is a multifactorial and complex process where the nutritional conditions, the human immune system, and the adaptability of the pathogen should be considered altogether to provide an effective treatment of the patient.

## 1. Introduction

*Candida* species are part of the normal flora of healthy individuals [1, 2] and are considered opportunistic pathogens as they colonize different tissues and cause systemic mycosis when the immune system of the host is depressed [3]. The commensal-pathogen shift requires an effective adaptation of* Candida* species to a particular environment in the human host in order to colonize it [4, 5]. It has been observed that the carbon source, which varies in the different tissues, is critical for colonization. For instance, the skin surface and mucosa contain low amounts of sugars, whereas the bloodstream may contain high levels of glucose [5–8]. This is important as* C. albicans* requires glucose or fructose to invade the genitourinary tract, whereas* C. glabrata* requires lactate to proliferate in the intestinal tract [8]. In addition to nutritional adaptation,* Candida* species express virulence factors that allow them to infect the host such as adhesins, yeast to hyphae switching, hydrolases, thigmotropism, and biofilm formation and to response to oxidative stress (OSR) [9–14]. Among these, biofilm formation is of special interest as this ability has been associated with high rates of morbility and mortality in hospitalized patients [2, 15, 16].* C. albicans* is the most frequently isolated pathogen from patients with disseminated candidiasis [17–19]. However, due to the indiscriminate use of wide-spectrum antibiotics [20–25] and medical implants, other non-*Candida albicans Candida* (NCAC) species, formerly considered as nonpathogenic, have emerged as frequent agents of human mycoses [13, 14]. Recurrent mycoses associated with NCAC are difficult to treat since some of these pathogens such as* C. glabrata, C. krusei,* and* C. tropicalis* are resistant to azole-based drugs [26–28]. On the contrary,* C. albicans* and* C. parapsilosis* are sensitive to fluconazole [29, 30]. Though the mechanisms of resistance of* Candida* species have not been completely elucidated, an attractive hypothesis is that biofilm formation on the surface of medical implants may confer protection to the pathogen [2]. This is due to the fact that cells in a biofilm community are irreversibly bound to a surface (sessile cells) and frequently embedded in an exopolymeric matrix, exhibiting distinct properties as compared to planktonic cells [31–33]. Sessile cells are highly resistant to antifungals and to the host mechanisms of defense [2, 31, 34–40], which makes biofilms a permanent source of infection as they are not only resistant to antifungals but also to the reactive oxygen and nitrogen species (ROS, RNS) produced during respiratory burst in human phagocytes. In line with this notion, it has been demonstrated that enzymes involved in H_2_O_2_ detoxification such as superoxide dismutase and catalase increase in OSR in biofilm cells of* Candida* spp. and* Escherichia coli* [41]. Though the ability of some* Candida* species to form biofilms on various medical implants has been evaluated as well as their OSR and adaptation to nutrients present in the target tissue [13, 37], it is however important to investigate how the carbon substrate impacts on biofilm formation by different* Candida* species and how sessile cells respond to antifungals and oxidative stress. These combined analyses will contribute to a better comprehension of the processes that take place in an immunocompromised patient. Here, we evaluated how* C. albicans* and three NCAC adapt to media containing different amounts of glucose during biofilm formation as well as the responses of sessile cells to antifungals, silver nanoparticles (AgNPs), and oxidative stress.

## 2. Materials and Methods

### 2.1. Strains and Culture Conditions

The strains of* C. albicans*,* C. glabrata*,* C. krusei*, and* C. parapsilosis* used in this study are clinical isolates from blood cultures of the collection of Departamento de Microbiología, ENCB-IPN, México, and were kindly donated by Professor M. A. Martínez Rivera. Yeast strains were cultured on yeast peptone (YP (yeast extract): 1%; peptone: 2%) or aminoacid-free yeast nitrogen base (YNB). YP and YNB media were supplemented with 0, 0.2, or 2% glucose (Sigma-Aldrich, USA), and 2% agar was added to solidify the media [42].

### 2.2. Biofilm Formation

All four* Candida* species were grown in YPD/2% for 48 h at 28°C. To promote biofilm formation, yeasts were harvested by low-speed centrifugation and resuspended in 100 *μ*L of the different media to a cell density of OD_600 nm_ 1.0. Each sample was placed into a well of flat-bottomed 96-well microtiter plates (Nunc, Nalgene) and incubated for 12, 24, or 48 h at 37°C. After biofilm formation, planktonic cells were discarded by washing the wells four times with sterile 10 mM calcium chloride (CaCl_2_, Sigma-Aldrich) prepared in phosphate-buffered saline, pH 7.2 (PBS, Sigma-Aldrich). Metabolic activity of sessile cells adhered to the plastic surface (biofilm) was measured using XTT [2,3-bis(2-methoxy-4-nitro-5-sulfophenyl)-2H-tetrazolium-5-carboxanilide] (Sigma-Aldrich), which is reduced by mitochondrial dehydrogenase to a water-soluble formazan that results in a colorimetric change [43]. Accordingly, 100 *μ*L of XTT-menadione (0.1 mg/mL XTT, 1 *μ*M menadione, Sigma-Aldrich) was added to each well and plates were incubated at 37°C. After 105 min, the XTT-derived formazan was measured at 492 nm using a microtiter plate reader (Varioskan Flash, Thermo Scientific). The same assay was used to determine the viability of sessile cells after exposure to different concentrations of H_2_O_2_ (0, 50, and 150 mM for* C. albicans *and* C. glabrata *and 0, 300, and 1500 mM for* C. krusei *and* C. parapsilosis*) or menadione (0, 2.4 and 8.4 mM for the four* Candida *species). Independent assays were done in triplicate.

### 2.3. Scanning Electron Microscopy (SEM)

Sterile polystyrene discs (0.7 cm in diameter) were placed in wells of 24-well tissue culture plates (Corning, USA). To form biofilms, 300 *μ*L of each* Candida* culture (OD_600 nm_ 1.0) was layered over the discs and plates were incubated at 37°C. After 48 h, free and nonadhered cells were eliminated by washing thrice with 10 mM CaCl_2_ and cells in biofilms were fixed for 3 h at room temperature with 4.0% (v/v) glutaraldehyde in 0.1 N sodium-cacodylate buffer, pH 7.4 (Sigma-Aldrich). Fixed cells were dried for 6 h in a Tousimis Auto Samdri 815 Critical Point Dryer, and dry samples were covered with a layer of colloidal gold and observed with a Leica F-420 model Sigma scanning electron microscope. Images were taken using a normal electrode SE detector at 10 kV under high vacuum conditions and at a working distance of 4 mm.

### 2.4. Assay of Antifungal Susceptibility

The effect on antifungals on the four* Candida* species was determined on planktonic (logarithmic or stationary phases) and sessile (biofilm) cells. For cells in logarithmic phase, fresh YPD medium was inoculated to a final density of OD_600 nm_ 0.03 and cells were allowed to grow at 28°C until they reached an OD_600 nm_ 0.1. For assays with cells in stationary phase, cultures were incubated for 48 h at 28°C and then diluted to a final density of OD_600 nm_ 0.1 in sterile deionized water. To analyze sessile cells, 100 *μ*L of sterile deionized water was added to each prewashed well and the cells were mechanically detached using a sterile spatula. The suspension of detached sessile cells was adjusted to an OD_600 nm_ 0.1. Sensitivity of both cell types to antifungals was determined by two methods: (a) broth microdilution assay according to the Clinical and Laboratory Standards Institute (CLSI) and (b) exponential dilutions of cells. Fluconazole, ketoconazole, itraconazole, and miconazole (Sigma-Aldrich) were used in both methods at final concentrations ranging from 0 to 256 *μ*g/mL. Antifungals were prepared in dimethyl sulfoxide (DMSO, Sigma-Aldrich) whose final concentration in all assays did not exceed 1%. In the first method, yeast cells were inoculated in Petri dishes containing solid Mueller Hinton or Sabouraud media (both from Becton-Dickinson). Sterile filter paper discs (6 mm in diameter, Whatman) were placed over the inoculated media and 4 *μ*L of aliquots containing different concentrations of antifungals (0.05, 0.18, 0.62, 2.1, 6.9, 23.0, 76.8, and 256 *μ*g/mL) was added on the disc surface and plates were incubated at 37°C. After 24–36 h, growth was inspected to determine the minimum inhibitory concentration (MIC). MIC values refer to the concentration of antifungal required to inhibit the visible growth of each* Candida* species. Photographs were taken with a Syngene Gene Genius Bio Imaging system. In the second method, suspensions of planktonic and sessile cells (OD_600 nm_ 0.1) were divided into equal aliquots that were incubated with antifungals at the concentrations indicated above and shaken at 37°C. After 90 min, cell density was readjusted to OD_600 nm_ 0.1 and exponential dilutions were prepared in 96-well plates and each dilution was spotted onto Mueller-Hinton agar plates. These were incubated at 37°C and growth was inspected after 48 h.

### 2.5. Assay of Susceptibility to AgNPs

Cultures of planktonic (logarithmic or stationary phase) and sessile cells were adjusted to OD_600 nm_ 0.1 and inoculated in Petri dishes containing Mueller-Hinton agar medium. Filter paper discs with antifungals were placed over the medium as described above and each disc received 20 *μ*L of AgNPs prepared according to Solomon et al. [44]. Plates were incubated at 28°C and photographs were taken after 24–36 h with a Syngene Gene Genius Bio Imaging system. Experiments were carried out in triplicate.

### 2.6. Assay of Susceptibility to H_2_O_2_ and Menadione

Equal aliquots of planktonic and sessile cells (OD_600 nm_ 0.5) were exposed to increasing concentrations of H_2_O_2_ or menadione (Sigma-Aldrich) and incubated with shaking at 28°C. After 90 min, the cell density of aliquots of treated cells was readjusted to OD_600 nm_ 0.5 and used to prepare exponential dilutions in 96-well plates. Each dilution was then spotted onto YPD plates and incubated at 28°C for 48 h. Photographs were taken with a Syngene Gene Genius Bio Imaging system. Experiments were carried out in quadruplicate.

### 2.7. Statistical Analysis

In order to maximize statistical power (minimum number of comparisons), all temporal data were summarized by using the area under the curve (AUC). This was computed for each set of data corresponding to all time points of one trial. The AUC data were analysed with a two-way ANOVA test, followed by a Bonferroni post hoc test (*α* = 0.05, ^*^ *P* < 0.05, ^**^ *P* < 0.01, and ^***^ *P* < 0.001). The AUC computation (trapezoid rule) and the statistical analysis were performed with the GraphPad Prism software (Graphpad, USA).

## 3. Results

### 3.1. Biofilm Formation by* Candida* Species under Different Nutritional Conditions

**Figure 1:**
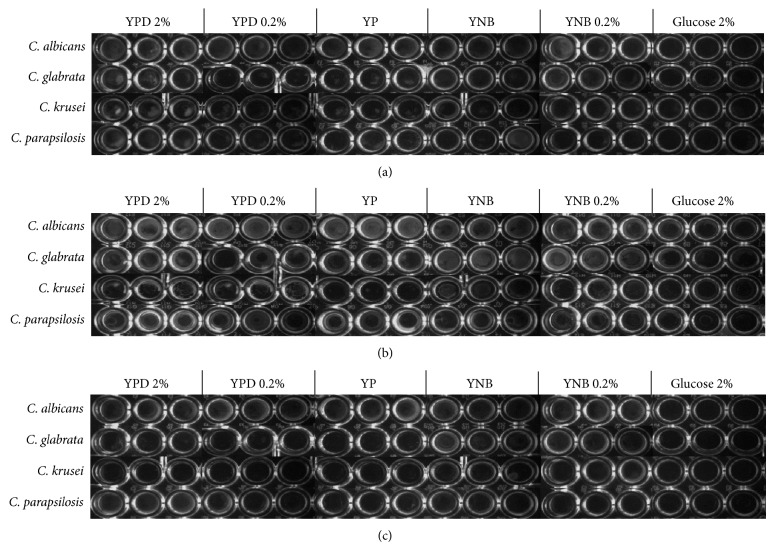
Biofilm formation by* C. albicans*,* C. glabrata*,* C. krusei*, and* C. parapsilosis*. Yeast cells grown as described in Materials and Methods were harvested by low-speed centrifugation and suspensions were adjusted to a final density of OD_600 nm_ 1.0 in 100 *μ*L of either YP or YNB media supplemented or not, with 0.2 or 2% glucose. Cells were placed in wells of flat-bottomed 96-well microtiter plates (Nunc, Nalgene) and incubated at 37°C. After 12 h (a), 24 h (b), and 48 h (c), biofilm formation was assessed and planktonic cells were removed by washing the wells with sterile 10 mM calcium chloride. Plates were photographed with a Syngene Gene Genius Bio Imaging system.

**Figure 2:**
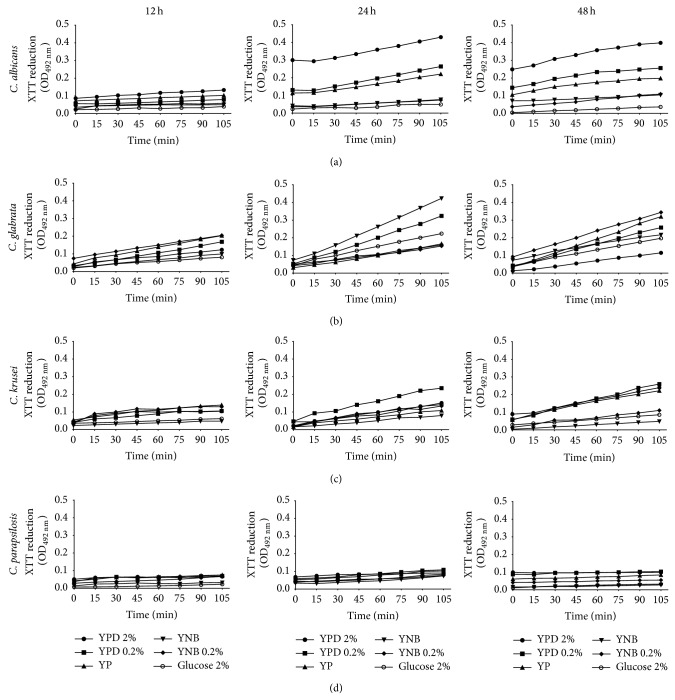
Temporal dynamics of sessile cells at 12, 24, and 48 h. The viability of the four* Candida* species was evaluated by measuring the amount of sessile cells with XTT in six nutritional environments. The temporal dynamics of the amount of cells was evaluated at eight consecutive time points (0, 15, 30, 45, 60, 75, 90, and 105 min) after time lapses of 12, 24, and 48 h. The nutritional environments were YP, YPD/0.2%, YPD/2%, YNB, YNB/0.2%, and YNB/2%. Plots show the mean value at each time point (mean of 3 assays) of XTT reduction.

**Figure 3:**
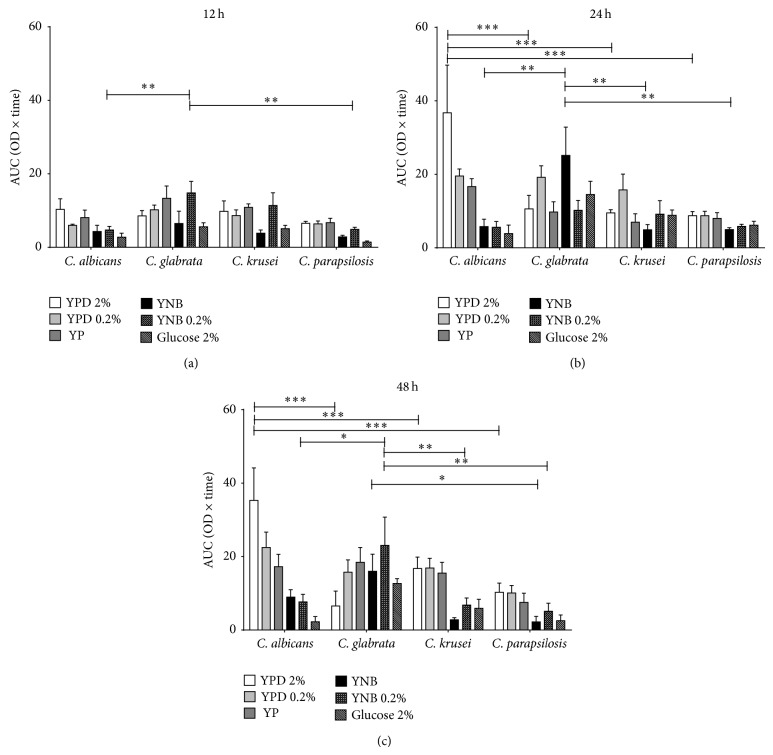
Area under the curve (AUC) of the amount of sessile cells. Bars represent the mean value (±SD) of the area under the curve (trapezoidal rule), which was computed for the OD values of eight consecutive time points (0, 15, 30, 45, 60, 75, 90, and 105 min) at intervals of 12, 24, and 48 h (panels a, b, and c, resp.). For comparison among* Candida* species and nutritional environments, at each elapsed interval, a two-way ANOVA was performed, followed by a Bonferroni post hoc test (*α* = 0.05, ^*^ *P* < 0.05, ^**^ *P* < 0.01, and ^***^ *P* < 0.001). The nutritional environments were YP, YPD/0.2%, YPD/2%, YNB, YNB/0.2%, and YNB/2%.

To evaluate their ability to adapt during biofilm formation, the four pathogenic* Candida* species were grown in two different media supplemented with different glucose concentrations. After 12 h,* C. albicans* formed biofilms in YPD/2%,* C. glabrata* in YNB and YNB/0.2%,* C. krusei* in YP, and* C. parapsilosis* in both YPD/0.2% and YPD/2% (Figure 1(a)). After 24 h,* C. albicans* formed biofilms in YP, YPD/0.2%, and YPD/2% and* C. glabrata* in YNB and YNB/0.2%, while* C. krusei* and* C. parapsilosis* did so in YPD/0.2% and YPD/2% (Figure 1(b)). Results obtained after 48 h (Figure 1(c)) revealed that* C. albicans* and* C. glabrata* formed biofilms under the same conditions observed after 24 h, while* C. krusei* and* C. parapsilosis* preferred YNB and YP, respectively (Figures 1(b) and 1(c)). It was observed that cells were not able to form mature biofilms after 12 h probably because this corresponds to the time required for adherence to the biomaterial and the start of cell increment. To confirm that cells in wells were not just adhered to polypropylene but they represented true biofilms, sessile cells were quantified by the XTT-based method as described previously. For all species, the number of sessile cells was very low after 12 h, but it significantly increased after longer times of incubation (Figure 2). It was also observed that* C. albicans* formed biofilms after 24 and 48 h in YPD/2% and* C. glabrata* in YNB and YNB/0.02%, whereas* C. krusei* and* C. parapsilosis* did so in YPD/0.2% (Figure 2). XTT reduction quantifies sessile cells of the four species of* Candida* in the different media and it is therefore possible to consider both factors, that is, media and species. This comparison allowed us to identify the ideal media for each species, that is, the one that results in easy adaptation and consequently efficient biofilm formation. Each assay consisted of eight time lapses between 0 and 105 min and six media. The temporal dynamics of the XTT assay was summarized by computing the area under the curve (trapezoidal method). This allows data reduction where each time interval (12, 24 and 48 h) is represented by six values for each species. Comparison of media and species was carried out by a two-way ANOVA test with *α* = 0.05 followed by a post hoc Bonferroni test. The highest numbers of sessile cells after 12 hours of incubation were obtained in YPD/2% (*C. albicans*); in YNB/0.2% (*C. glabrata* and* C. krusei*); and in YP, YPD/0.2%, and YPD/2% (*C. parapsilosis*) (Figure 3(a)). After 24 and 48 h,* C. albicans* was able to develop a biofilm in YPD/2%, while* C. glabrata* formed it in YNB and YNB/0.2% (Figures 3(b) and 3(c)).* C. krusei *formed biofilms in YPD/0.2% after 24 h (Figure 3(b)) and in YP, YPD/0.2%, andYPD/2% after 48 h. On its part,* C. parapsilosis* developed biofilms in YP, YPD/0.2%, and YPD/2% after 24 h (Figures 3(b) and 3(c)). In contrast with* C. albicans*,* C. krusei* and* C. parapsilosis* which preferred a rich medium such as YPD,* C. glabrata* preferred YNB (Figures 2 and 3).

Although data obtained in this study support the notion that each* Candida* species requires specific nutritional conditions to form biofilms, it may be necessary to include a significant number of strains of each* Candida* species in similar future studies to evaluate whether a correlation exist* in vivo* between the physiological state of the patient and the pathogens. Yet, our observations on the effect of nutritional conditions* in vitro* support the notion that,* in vivo,* each* Candida *species differentially adhere and colonize a physiological niche, thus resulting in the onset of infection.

### 3.2. Analysis of Biofilms by SEM

**Figure 4:**
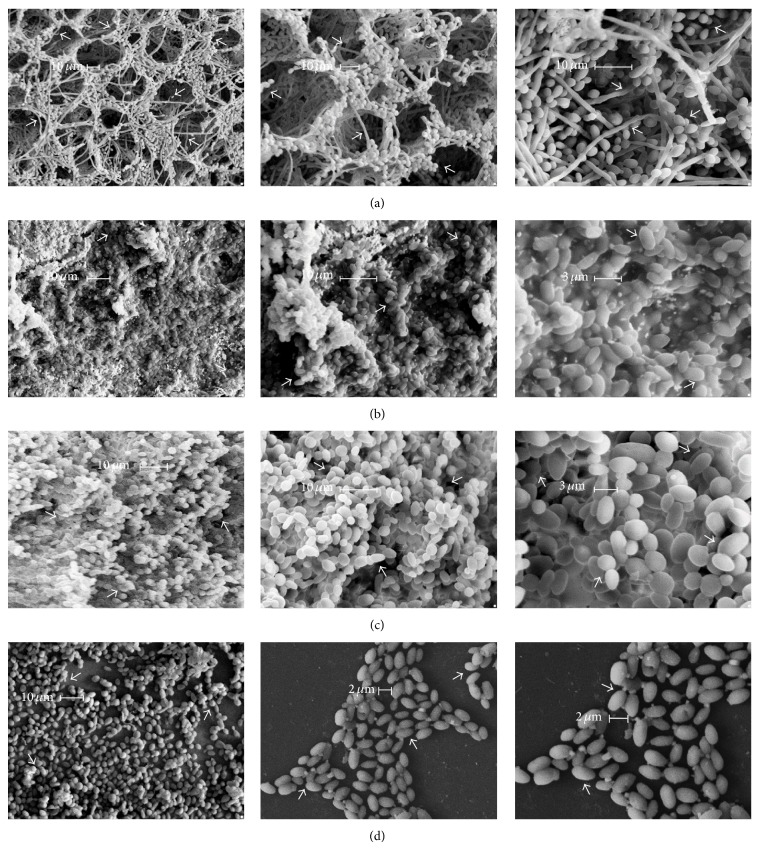
Biofilms formed by* Candida* species as observed by SEM. Samples were observed with a LEICA F-420 SIGMA model and scanning electron microscope using a normal electrode SE detector at 10 kV under high vacuum conditions and at a working distance of 4 mm. (a)* C. albicans* in YPD/2%; (b)* C. glabrata* in YNB/0.2%, (c)* C. krusei* in YPD/2%, and (d)* C. parapsilosis* in YPD/2%. The scale bar is indicated for each microphotography. White arrows indicate yeast, hyphae, or pseudohyphae cells.

Biofilms formed by the four* Candida* species in the conditions described above were observed by SEM (Figure 4).* C. albicans* biofilms consisted of yeast, pseudohyphae, and hyphae cells immersed in a highly organized structure where pores with diameters in the range of 20–40 *μ*m are clearly evident as well as the presence of an exopolymeric matrix, as has been observed by other authors (Figure 4(a)) [45–47]. The biofilm formed by* C. glabrata *contained yeast cells only and it also exhibited pores of about 8.5 *μ*m and a less abundant exopolymeric material as compared to* C. albicans* (Figure 4(b)). The* C. krusei* biofilm also contained yeast cells, pores of about 3.0 *μ*m, and residual amounts of the exopolymeric substance (Figure 4(c)) as compared to* C. albicans* and* C. glabrata* (Figures 4(a) and 4(b)). Finally,* C. parapsilosis *was unable to form a biofilm and neither a considerable cell density nor an exopolymeric product (Figure 4(d)). These observations suggest that nutrients, present in the medium, as well as the ability of each species to adapt to it, influence the formation, composition, and structure of biofilms by* Candida* cells.

### 3.3. *In Vitro* Effect of Azole-Based Antifungals Either Alone or Combined with AgNPs on Planktonic and Sessile Cells of* Candida* Species

**Figure 5:**
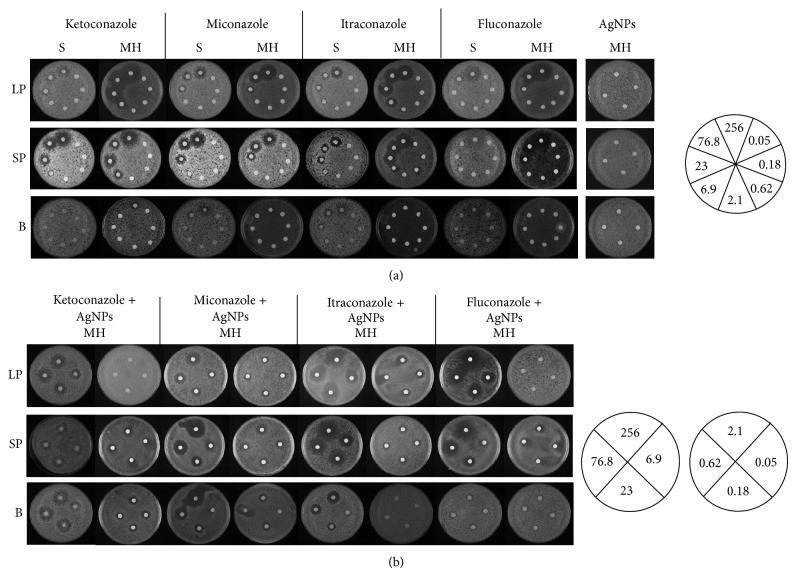
Susceptibility of sessile and planktonic cells to azole-derived antifungals and AgNPs. Planktonic (either from logarithmic or stationary phase) and sessile cells of* C. albicans* were diluted to an OD_600 nm_ 0.1 with sterile deionized water. (a) Aliquots of dilutions were incubated at 37°C in the presence of increasing concentrations of the indicated antifungals or (b) antifungals plus AgNPs. After 90 min, cells were pelleted by low-speed centrifugation and resuspended in sterile deionized water to an OD_600 nm_ 0.1. LP, logarithmic phase; SP, stationary phase; B, biofilm; AgNPs, silver nanoparticles; S, Sabouraud medium; MH, Mueller-Hinton medium.

The functionality of biofilms formed by each* Candida* species was evaluated by determining their responses to azole antifungals either alone or in combination with AgNPs. In addition, two culture media such as Sabouraud (S) and Mueller-Hinton (MH) were used to compare the susceptibility to antifungals under different growth conditions. For these assays, yeast planktonic cells in logarithmic (LP) or stationary (SP) phases were used as control as it has been demonstrated that they are more susceptible than biofilm sessile cells to antifungals [34–36]. Results shown in Figure 5 and Table 1 indicate that LP cells of* C. albicans* exhibit significant differences in resistance; accordingly, they resisted up to 256 *μ*g ketoconazole/mL in S, whereas this concentration decreased to only 2.1 *μ*g/mL in MH. However, in both media* C. albicans* was equally sensitive to miconazole (6.9 *μ*g/mL) and itraconazole (2.1 *μ*g/mL). On the other hand, this pathogen was over 3-fold more susceptible to fluconazole in S than in MH (23* versus* 9 *μ*g/mL). SP yeast cells of* C. albicans* were comparably susceptible toketoconazole (2.1 *μ*g/mL) and miconazole (6.9 *μ*g/mL) in both media, whereas they were more resistant to itraconazole in S than in MH (6.9* versus* 2.1 *μ*g/mL). This response contrasts with the resistance shown to fluconazole, namely, up to 256 *μ*g/mL in S whereas only 6.9 *μ*g/mL in MH. Noticeably, sessile cells of* C. albicans* were resistant to the four tested antifungals up to concentrations of 256 *μ*g/mL in both S and MH media (Figure 5(a); Table 1). Sessile cells of the other three* Candida* species were in general more resistant to antifungals than planktonic cells (Table 1).

**Figure 6:**
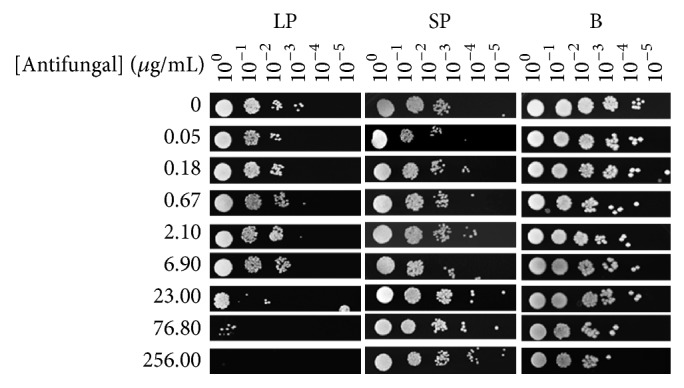
Susceptibility of planktonic and sessile cells to antifungals. Samples of cultures of* C. albicans* in stationary phase were diluted to an OD_600 nm_ 0.1 with sterile deionized water. Aliquots of dilutions were incubated at 37°C in the presence of the indicated concentrations of antifungals_. _After 90 min, water was removed by low-speed centrifugation and the corresponding cell pellets were resuspended in sterile deionized water to an OD_600 nm_ 0.1. Samples of these suspensions were exponentially diluted in 96-well plates and each dilution was spotted onto Mueller-Hinton agar plates that were incubated at 37°C. Growth was inspected after 48 h. LP, logarithmic phase; SP, stationary phase; B, biofilm.

To determine whether sessile cells also resisted high concentrations of an antimicrobial agent, the combined effect of antifungals and AgNPs was determined. Mock incubations containing AgNPs only were run in parallel. Surprisingly, we observed that both sessile and planktonic cells were able to grow in the presence of the nanoparticles (Figure 5(a)) indicating that at the used concentration AgNPs are not toxic to the four* Candida* species. It has been described that microbicidal effect of AgNPs depends on a number of factors such as the size and form of nanoparticles, their chemical composition, the coating, and potential surface charge and concentration [48]. Results shown in Table 1 indicate that sessile but not planktonic cells of the four species were able to grow in the presence of antifungals and AgNPs. For instance, biofilm-associated cells of* C. albicans* grew in the presence of up to 23 *μ*g/mL itraconazole-AgNPs, whereas corresponding planktonic cells were susceptible at 2.1 *μ*g/mL (Figure 5(b)). Sessile cells of the other species were similarly resistant to antifungals (Table 1). In the assay of susceptibility by serial dilutions, cells are exposed to antifungals for a short period of time (90 min in this study). Representative results obtained with one* Candida* species are depicted in Figure 6. LP planktonic cells of* C. albicans* resisted up to 6.9 *μ*g/mL ketoconazole, while those from SP resisted up to 256 *μ*g/mL, which is the same as for sessile cells (Figure 6, Table 2). Both planktonic and sessile cells of* C. glabrata*,* C. krusei*, and* C. parapsilosis* resisted up to 256 *μ*g/mL fluconazole and itraconazole, but sessile cells were more resistant to ketoconazole and miconazole (Table 2). These results are in line with those observed in the other assay (longer, chronic exposure, Figure 5 and Table 1) where sessile cells were more resistant and* Candida* species were able to grow in the presence of high concentrations of fluconazole and itraconazole (Figure 6, Table 2).

### 3.4. Susceptibility of Sessile Cells of* Candida* Species to Oxidative Stress

**Figure 7:**
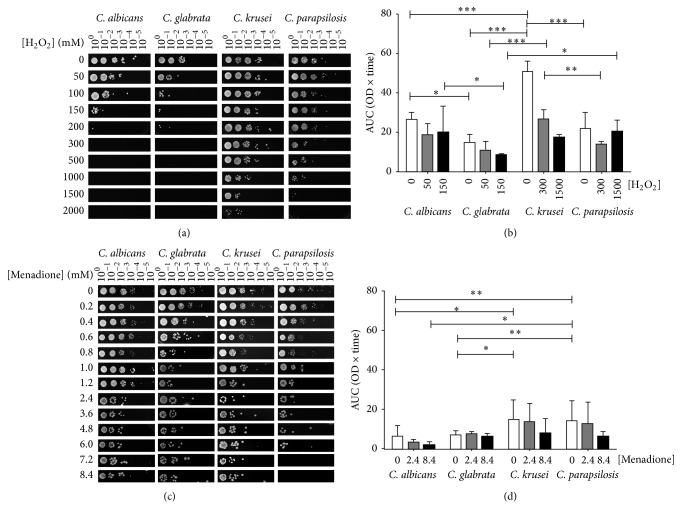
Response of sessile cells of* Candida* species to H_2_O_2 _and the O_2_^•−^ ion. (a, c) Sessile cells of the four* Candida *species were diluted to an OD_600 nm_ 0.5 with sterile deionized water. Aliquots of dilutions were incubated at 28°C in the presence of the indicated concentrations of (a) H_2_O_2 _and (c) menadione. After 90 min, water was removed by low-speed centrifugation and the corresponding cell pellets were resuspended in sterile deionized water to an OD_600 nm_ 0.5. Samples of these suspensions were exponentially diluted in 96-well plates and each dilution was spotted onto YPD plates that were incubated at 28°C. Growth was inspected after 48 h. (b, d) Bars represent the mean value (±SD) of the area under the curve (trapezoidal rule) which was computed for the OD values of eight consecutive time points (0, 15, 30, 45, 60, 75, 90, and 105 min) at intervals of 12, 24, and 48 h. For comparison among* Candida* species and H_2_O_2 _(b) or menadione (d), at each elapsed interval, a two-way ANOVA was performed, followed by a Bonferroni post hoc test (*α* = 0.05, ^*^ *P* < 0.05, ^**^ *P* < 0.01, and ^***^ *P* < 0.001).

It has been described that biofilm-associated cells are resistant to ROS and RNS produced during the phagocytic respiratory burst in the human host. Here,* C. albicans* and* C. glabrata* were able to grow in the presence of 150 mM H_2_O_2_ in contrast to the 10-fold higher resistance shown by* C. krusei* and* C. parapsilosis* (Figure 7(a)). The case of* C. glabrata* is of special interest as we have shown that in both LP and SP this species resists higher concentrations of this oxidant than* C. albicans* [49, 50].

On this background, the number of viable sessile cells after exposure of* Candida* species to H_2_O_2_ was determined. Results are shown in Figure 7(b). The amount of viable cells decreased when* C. albicans* and* C. parapsilosis* were exposed to 50 and 300 mM H_2_O_2_, respectively; however, the number of cells increased after exposure of both species to a higher concentration of the oxidant. In regard to* C. glabrata* and* C. krusei*, cell viability decreased as a function of higher concentrations of oxidant.

With respect to superoxide ion, results in Figure 7(c) indicate that* C. albicans, C. glabrata, *and* C. krusei* were able to survive up to a concentration of 8.4 mM menadione, thus being the most resistant species to ion O_2_^•−^. It is possible that this pathogen is able to withstand an even higher concentration of this oxidant; however, we failed to determine the minimum lethal concentration (MLC) as menadione is not soluble in DMSO at higher concentrations. In contrast to the other three species,* C. parapsilosis* showed less resistance and was viable up to 6.0 mM menadione. As illustrated in Figure 7(d), its viability decreased as a function of higher concentrations of the oxidant (Figure 7(d)). These results are in accord with those obtained by the dilution assay method (Figure 7(c)).

## 4. Discussion

### 4.1. *Candida* Species Can Form Biofilms under Specific Nutritional Conditions

Formation of biofilms by* Candida* species is a major cause of the high rates of hospital morbility and mortality [32]. During this process, the organism must adapt to some particular conditions prevailing in the target tissue including the ROS generated inside the phagolysosome. Here, we demonstrated that* C. glabrata* can form biofilms under rather poor nutritional conditions and a low glucose concentration in contrast to the other three species that require a rich medium such as YPD (Figures 2 and 3). This is probably due to the fact that* C. glabrata* has adapted to these conditions to preferentially colonize some human tissues and not to compete for the same physiological niche with, for instance,* C. albicans.* This idea is in accord with the observation that* C. albicans* is mainly isolated from glucose-rich environments such as the bloodstream [4–6, 8, 51]. It is not then surprising that* C. albicans* is the species most frequently isolated from patients suffering diabetes mellitus (DM) [52–54]. This prevalence is probably due to the finding that glucose directly promotes the resistance of* C. albicans* to antifungals, a hypothesis confirmed by experiments with diabetic mice [55]. As a consequence, administration of glucose for long periods of time to hospitalized patients may also favour tissue colonization and invasive candidiasis [56].

### 4.2. Each* Candida* Species Forms Structurally Different Biofilms

The differences observed in this study among biofilms formed by* Candida* species (Figure 4) suggest that, apart from adapting to specific nutritional conditions, each of these pathogens has developed distinct mechanisms to form biofilms and this may be related to their ability to disseminate through the bloodstream and colonize different tissues of the human body [57]. We believe that substrate requirements of each species can influence the biofilm properties. In this regard, it has been shown that galactose affects adhesion of* Candida* to host cells [45]. As described in other reports,* C. albicans* produces an exopolymeric matrix that consists of polysaccharides, proteins, and signalling molecules that serve the organism to distribute nutrients and as a barrier for the diffusion of antifungals into the biofilm [2, 45–47]. It has been shown that formation of the characteristic structure of* C. albicans* biofilm depends on a delicate regulation of adhesins and mannoproteins by transcriptional factors such as Bcr1, Efg1, Ndt80, Rob1, and Brg1 [58–60].

In contrast,* C. glabrata*,* C. krusei*, and* C. parapsilosis* produce only small amounts of exopolymeric material as observed here and by other authors [43, 61, 62]. Nonetheless, sessile cells of these pathogens resist high concentrations of antifungals (Table 1). Thus, the exopolymeric material does not contribute significantly for the survival of NCAC against antifungals. Furthermore, these findings indicate that such resistant of NCAC to azole-based antifungals might be related mainly to (1) phenotypic changes which results in poor growth or nutrient limitation and (2) the expression of resistance genes induced by the contact with the host's cells.

### 4.3. Sessile Cells of* Candida* Are More Resistant Than Corresponding Planktonic Cells to the Combined Effect of Antifungals and AgNPs

Last years have witnessed a rapid increase in the development of anti-*Candida* drugs and consequently to a modification in the mechanisms of resistance of these pathogens to antifungals administered to patients [63–66]. In this context, it has been reported that patients with a newly implanted medical device respond well to an antifungal therapy but after some time they develop recurrent candidiasis [67] indicating that the pathogen has formed a biofilm with a higher resistance to the treatment [68]. In line with this idea, it has been demonstrated that biofilm-associated, sessile cells can resist high concentrations of antifungals [69, 70], as observed also in this study when sessile cells of the four species of* Candida* were compared with the corresponding planktonic cells. Mechanisms of resistance may depend on (i) changes in cell wall that interfere with drug assimilation, (ii) a decrease in the affinity of the drug for its target due to either overexpression or mutation of genes coding for enzymes in the ergosterol pathway such as 14*α*-lanosterol demethylase, encoded by the* ERG11* gene, which is the main target of fluconazole [71–75], and (iii) an increase in the activity of membrane transport proteins involved in drug efflux [65, 71, 72].

It was interesting to observe not only the fact that sessile cells of* Candida* are more resistant to antifungals but also the fact that they are able to grow in the presence of AgNPs indicating that cells in biofilms trigger mechanisms that enable them provide protection also from other antimicrobial agents.

We believe that these characteristics are the major cause of recurrent and invasive candidiasis in hospitalized and immunocompromised patients [68] that often result in fatal outcomes. We also noted that planktonic cells from the same growth phase exhibited differences in their susceptibility to antifungals depending on the culture medium, thus supporting the importance of adaptation to nutritional conditions on these responses.

Our data also indicate that both planktonic and sessile cells of the four* Candida* species require adaptation to the media in order to respond efficiently to the antifungals. Interestingly, once* Candida* has formed the biofilm, sessile cells are able to withstand high concentrations of an antifungal-AgNPs mixture regardless of the media. Moreover, the fact that* Candida* planktonic cells are not susceptible to AgNPs (Figure 5(a)) in contrast to the response to combined AgNPs and antifungals (Figure 5(b)) suggests that these pathogens have developed mechanisms that allow them to provide protection from certain toxic compounds such as nanoparticles (NPs) at certain concentrations [48] and also that these pathogens require some other factors such as antifungals to destabilize the cell wall so AgNPs can express their microbicidal effect (Figures 5(a) and 5(b)). Furthermore, our data also support the notion that sessile cells in contrast to planktonic cells possess mechanisms that allow them to resist antifungals either alone or combined with other microbicides. This is in agreement with reports in other organisms such as bacteria where it has been postulated that transport of NPs perturbs cell membrane functions resulting in alterations of proteins and DNA or production of ROS in response to their presence in the cytoplasm [48, 76]. So, for this to occur in* Candida,* it would be necessary that some compounds such as an antifungal alter the CW facilitating the access of NPs to the membrane to act as a microbicidal. In this regard, it has recently been described that AgNPs cannot permeate* Candida* cells [77]. Hopefully, this observation may help to choose other treatment alternatives such as miconazole, an azole very active against the four* Candida* species.

### 4.4. Susceptibility of* Candida* Species to H_2_O_2_ and the O_2_^•−^ Ion

The susceptibility of* C. albicans* and the resistance of* C. krusei* and* C. parapsilosis* to H_2_O_2_ (Figures 7(a) and 7(b)), regardless of the growth phase, are well-documented characteristics of these species [11, 49, 78]. We observed that sessile cells of* C. glabrata* are equally sensitive to this oxidant than* C. albicans*, which does not occur in the response of planktonic cells to oxidative stress [11, 49]. We have also shown that cells of* C. glabrata* either in logarithmic or stationary phases are more resistant to H_2_O_2_ than those of* C. albicans* [11, 49]. Nevertheless, even when sessile cells of* C. albicans* and* C. glabrata* are more sensitive to H_2_O_2_ than* C. krusei* and* C. parapsilosis*, they are the species that are most frequently isolated from hospitalized and immunocompromised patients.

Regarding the O_2_^•−^ ion, it has been demonstrated that resistance of planktonic cells of* C. albicans* to high concentrations of superoxide, in contrast to those* C. glabrata* and* C. krusei* which are more susceptible, is mostly due to its ability to detoxify this oxidant [12]. Here, we observed that sessile cells of* C. albicans* maintain the natural resistance to this superoxide, while those* C. glabrata*,* C. krusei*, and* C. parapsilosis* resist higher concentrations of O_2_^•−^ ion than corresponding planktonic cells (Figures 7(c) and 7(d)) [12, 50]. This is interesting as it indicates that biofilm formation favours the resistance of these pathogens to antifungals and ROS meaning that* in vivo* it can help their prevalence and dissemination in the human host.

## 5. Conclusions

To our knowledge, this is the first report on the relevance of nutritional conditions and glucose on the formation of biofilms by four species of* Candida*. In general, sessile cells are more resistant to high concentrations of antifungals and oxidative stress. This indicates that infection by these pathogens is a multifactorial process involving adaptation to particular conditions in target tissues and the human immune system, factors that should be considered during prevention and treatment of invasive mycosis. Daily administration of nutrients such as serum/glucose favours infections by most of the pathogenic species of* Candida*. The design and synthesis of new antifungals administered by short periods of time to prevent* Candida* from adaptation and thus formation of biofilms are the most desirable tasks to come up in the near future with a more efficient therapy.

## Figures and Tables

**Table:** (a) Aliquots of dilutions were incubated at 37°C in the presence of increasing concentrations of the indicated antifungals

Species	Ketoconazole (*µ*g/mL)						Ketoconazole (*µ*g/mL) + AgNPs			Miconazole (*µ*g/mL)						Miconazole (*µ*g/mL) + AgNPs		
	LP		SP		B		LP	SP	B	LP		SP		B		LP	SP	B
	S	MH	S	MH	S	MH	MH	MH	MH	S	MH	S	MH	S	MH	MH	MH	MH
*C. albicans *	256	2.1	2.1	2.1	256	256	6.9	0.05	0.05	6.9	6.9	6.9	256	256	6.9	0.05	0.05	0.05
*C. glabrata *	256	256	256	76.8	256	256	6.9	76.8	256	6.9	6.9	6.9	6.9	6.9	6.9	0.05	0.05	2.1
*C. krusei *	256	256	256	23	256	256	0.05	6.9	0.05	6.9	6.9	6.9	23	23	6.9	0.05	0.05	0.05
*C. parapsilosis *	76.8	6.9	6.9	76.8	6.9	6.9	6.9	0.05	6.9	76.8	23	23	76.8	76.8	76.8	0.05	0.05	6.9

**Table:** (b) antifungals plus AgNPs. After 90 min, cells were pelleted by low-speed centrifugation and resuspended in sterile deionized water to an OD_600 nm_ 0.1

Species	Fluconazole (*µ*g/mL)						Fluconazole (*µ*g/mL) + AgNPs			Itraconazole (*µ*g/mL)						Itraconazole (*µ*g/mL) + AgNPs		
	LP		SP		B		LP	SP	B	LP		SP		B		LP	SP	B
	S	MH	S	MH	S	MH	MH	MH	MH	S	MH	S	MH	S	MH	MH	MH	MH
*C. albicans *	23	6.9	256	6.9	256	256	0.05	0.05	0.05	2.1	2.1	6.9	2.1	256	256	0.05	0.05	6.9
*C. glabrata *	256	256	256	76.8	256	256	0.05	256	256	76.8	6.9	256	6.9	256	256	0.05	0.05	76.8
*C. krusei *	256	256	256	256	256	256	0.05	0.05	0.05	76.8	2.1	256	6.9	256	256	0.05	0.05	0.05
*C. parapsilosis *	256	76.8	256	256	256	256	6.9	256	256	2.1	2.1	6.9	2.9	6.9	6.9	0.05	23	2.1

LP, logarithmic phase; SP, stationary phase; B, biofilm; AgNPs, silver nanoparticles; S, Sabouraud medium; MH, Mueller-Hinton medium.

**Table 2:** Resistance of planktonic and sessile cells of *Candida* species to antifungals. Cultures in stationary phase of the four *Candida* species were diluted to an OD_600 nm_ 0.1 with sterile deionized water. Aliquots of dilutions were incubated at 37°C in the presence of the indicated concentrations of antifungals. After 90 min, water was removed by low-speed centrifugation and the corresponding cell pellets were resuspended in sterile deionized water to an OD_600 nm_ 0.1. Samples of these suspensions were exponentially diluted in 96-well plates and each dilution was spotted onto Mueller-Hinton agar plates that were incubated at 37°C. Growth was inspected after 48 h. LP, logarithmic phase; SP, stationary phase; B, biofilm.

Species	Fluconazole (*µ*g/mL)			Itraconazole (*µ*g/mL)			Ketoconazole (*µ*g/mL)			Miconazole (*µ*g/mL)		
	LP	SP	B	LP	SP	B	LP	SP	B	LP	SP	B
*C. albicans *	256	256	256	256	256	256	6.9	256	256	23	256	76.8
*C. glabrata *	256	256	256	256	256	256	23	76.8	256	6.9	76.8	256
*C. krusei *	256	256	256	256	256	256	23	256	256	76.8	256	256
*C. parapsilosis *	256	256	256	256	256	256	76.80	256	256	23	256	256
